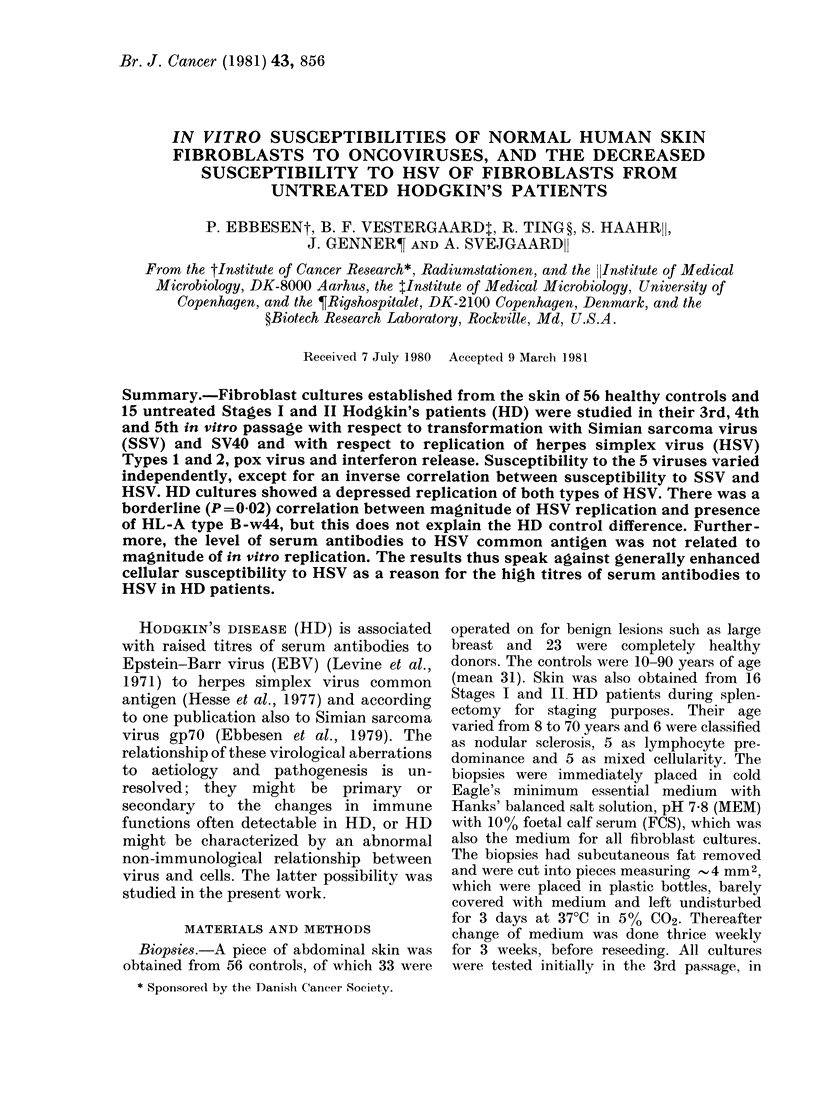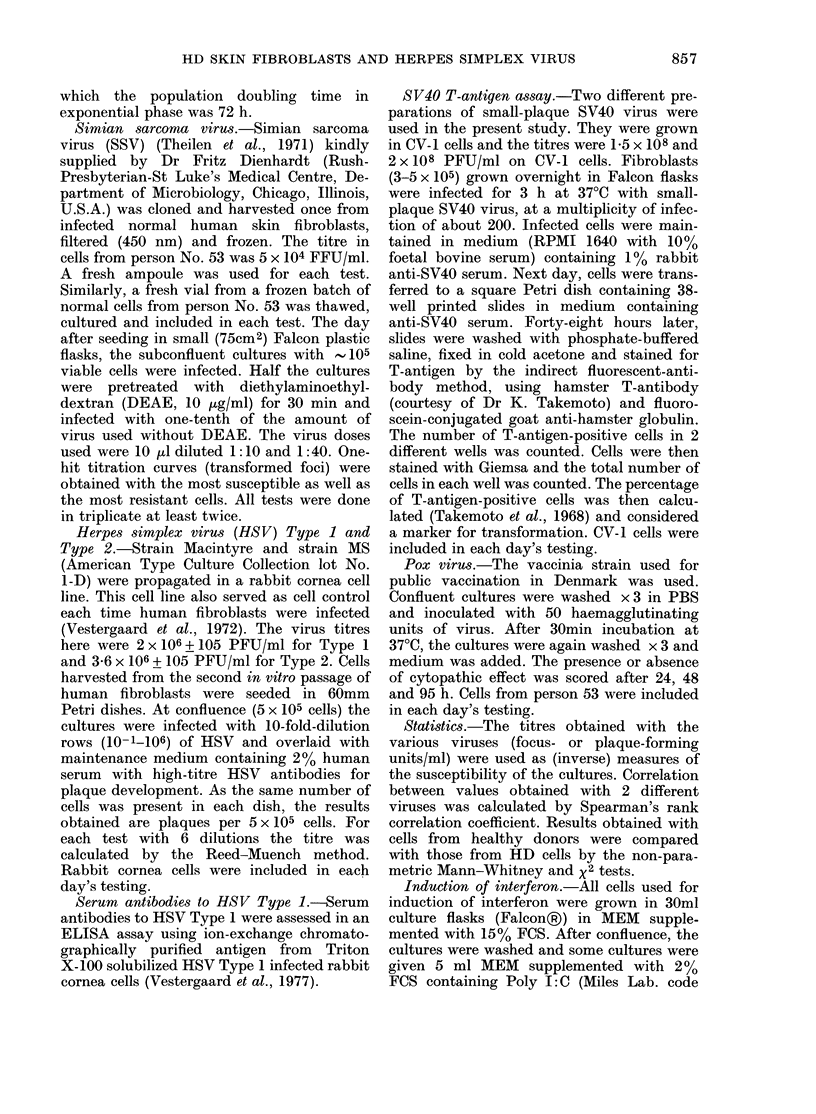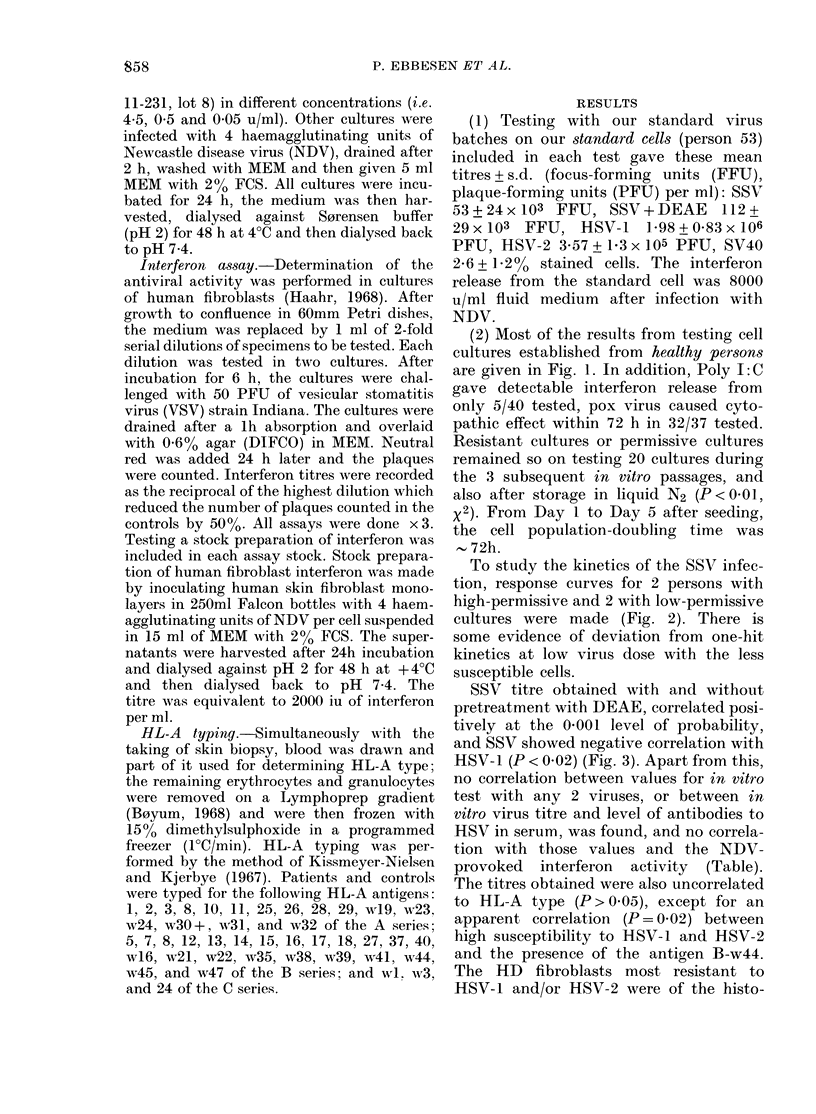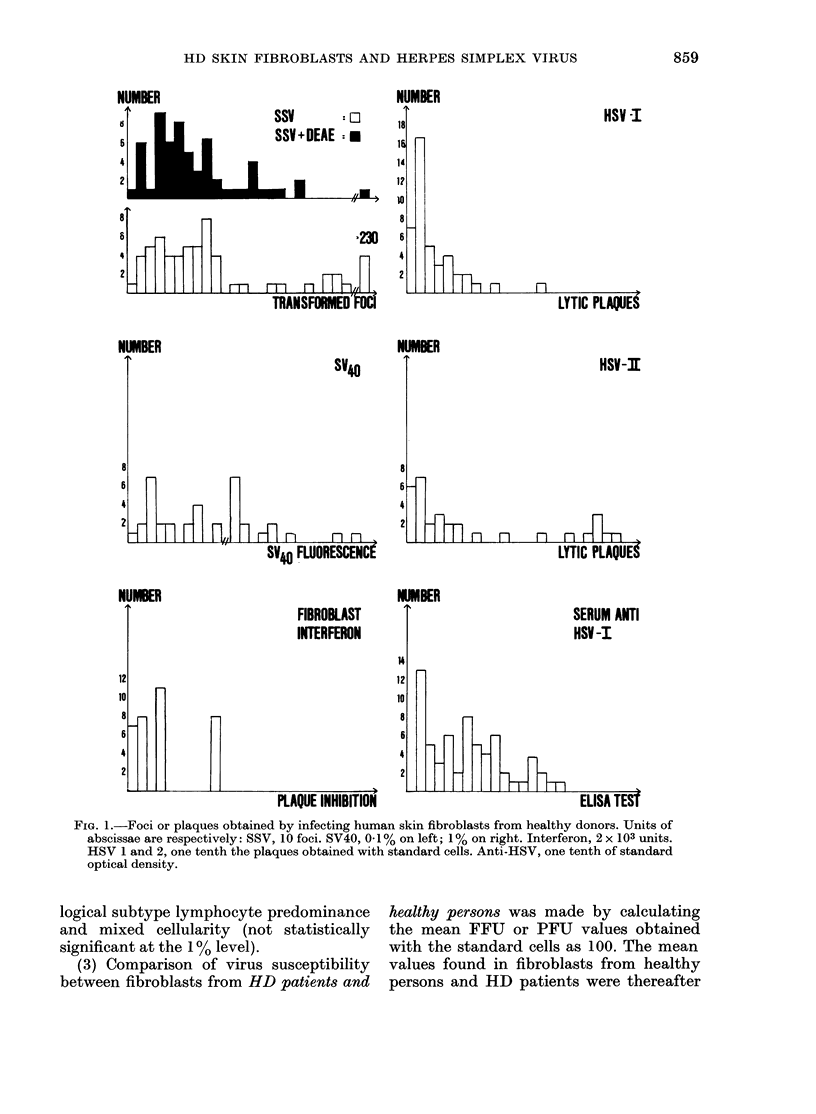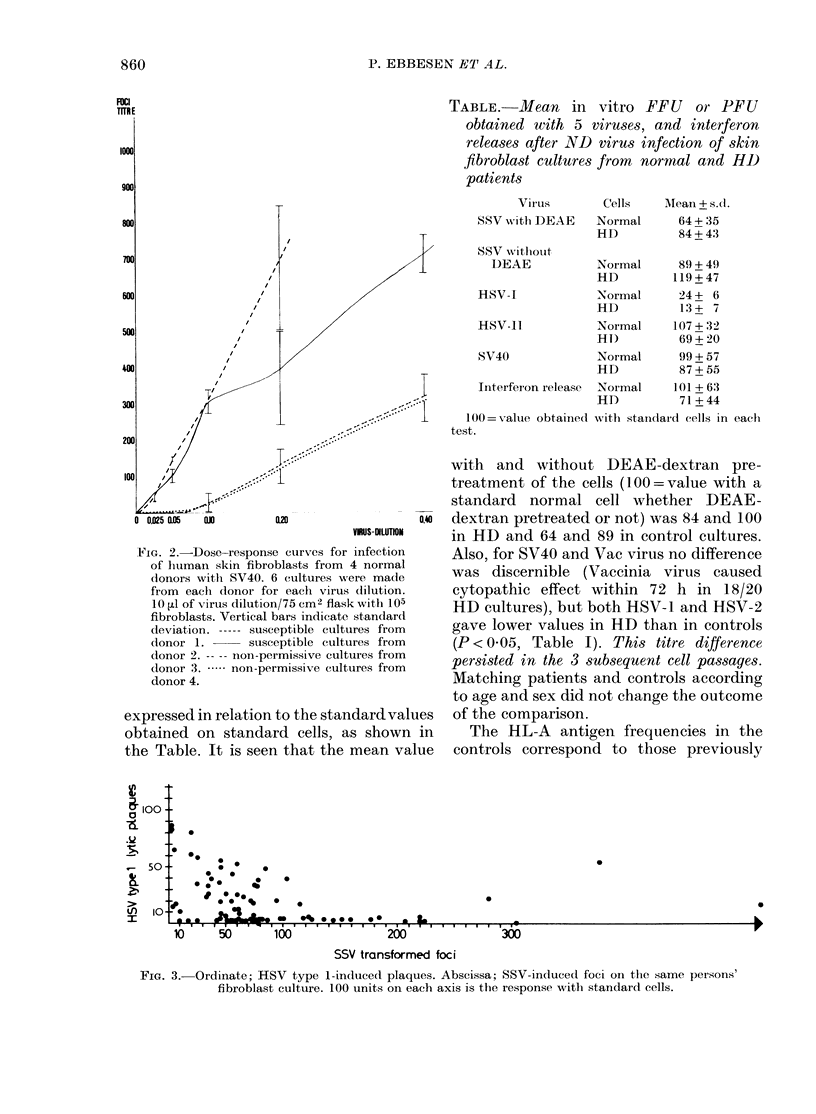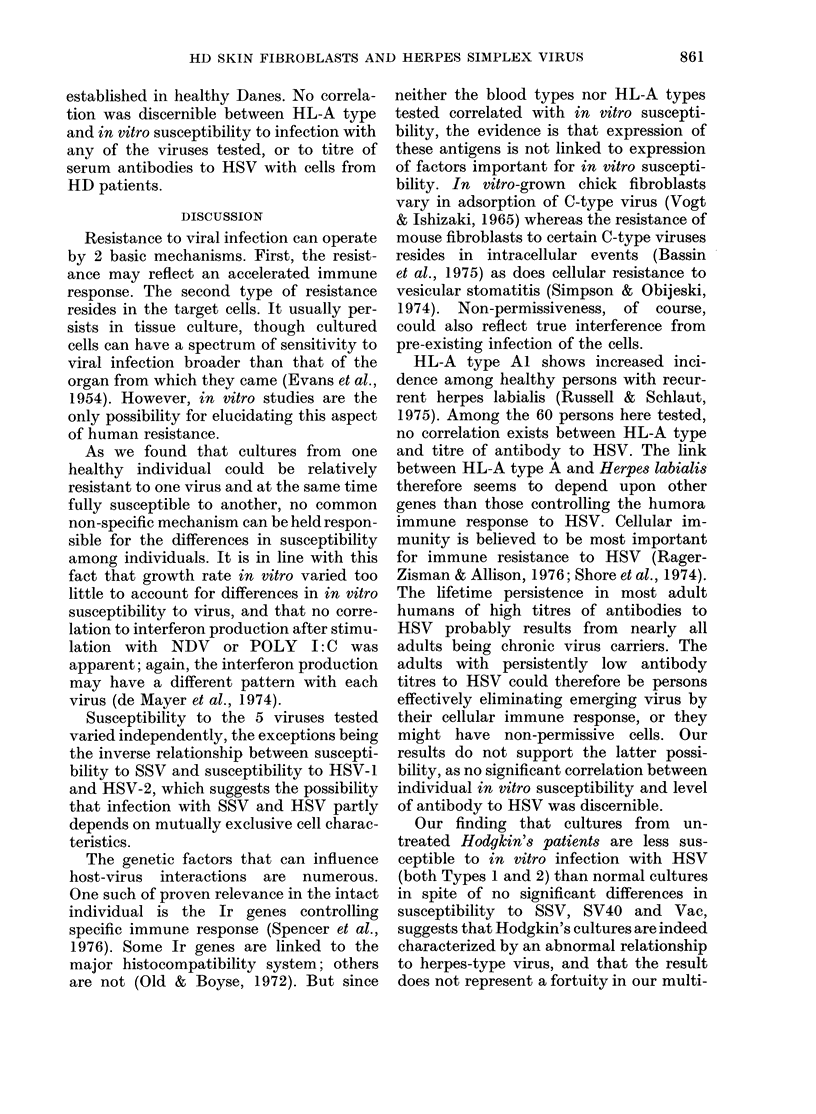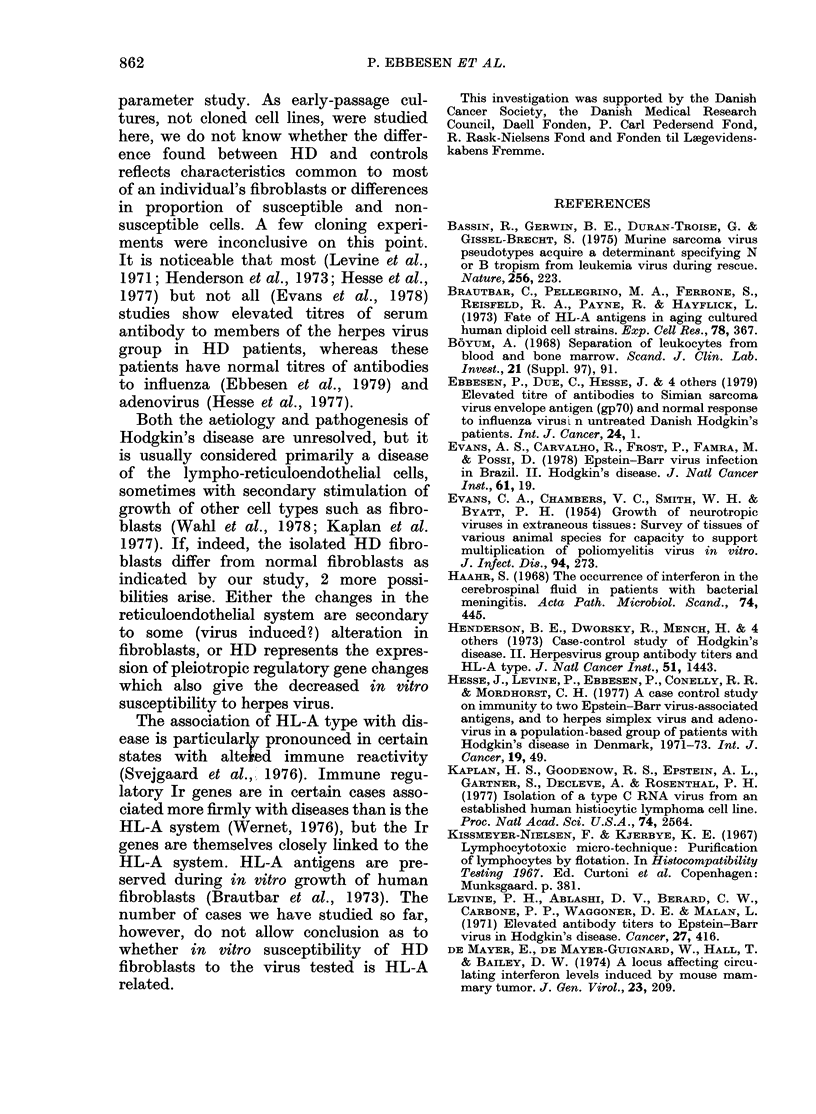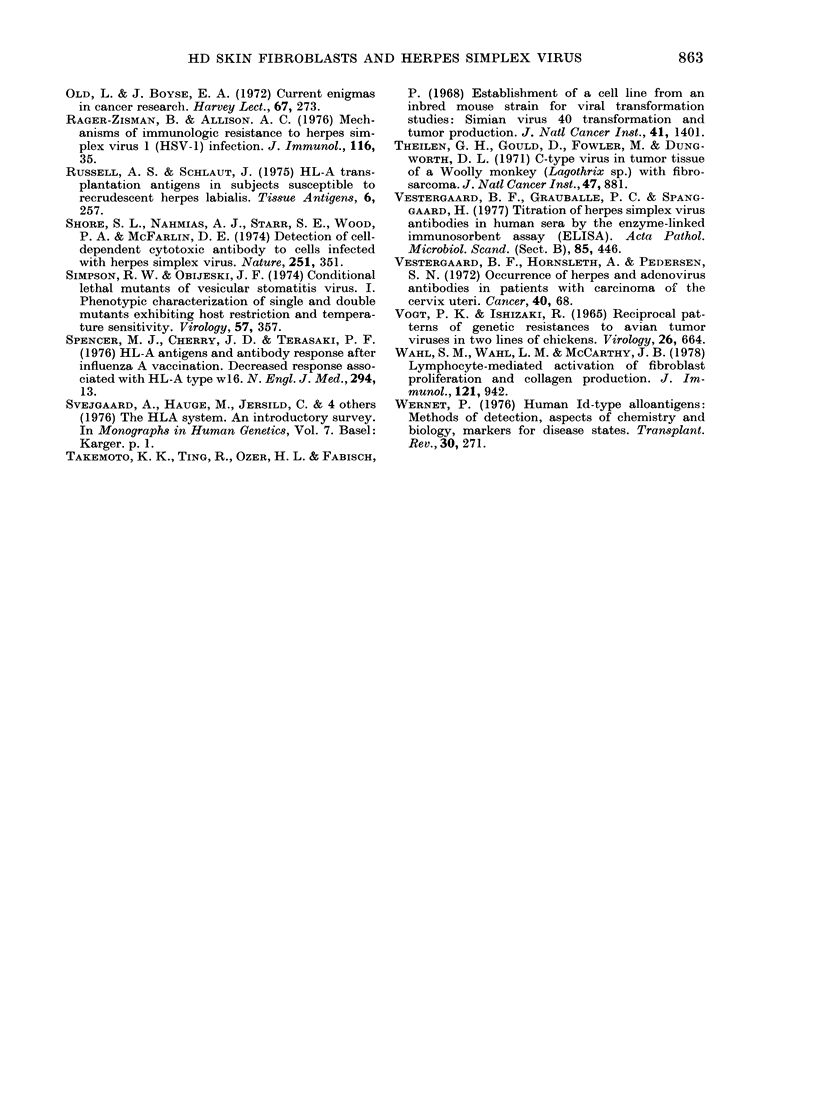# In vitro susceptibilities of normal human skin fibroblasts to oncoviruses, and the decreased susceptibility to HSV of fibroblasts from untreated Hodgkin's patients.

**DOI:** 10.1038/bjc.1981.125

**Published:** 1981-06

**Authors:** P. Ebbesen, B. F. Vestergaard, R. Ting, S. Haahr, J. Genner, A. Svejgaard

## Abstract

Fibroblast cultures established from the skin of 56 healthy controls and 15 untreated Stages I and II Hodgkin's patients (HD) were studied in their 3rd, 4th and 5th in vitro passage with respect to transformation with Simian sarcoma virus (SSV) and SV40 and with respect to replication of herpes simplex virus (HSV) Types 1 and 2, pox virus and interferon release. Susceptibility to the 5 viruses varied independently, except for an inverse correlation between susceptibility to SSV and HSV. HD cultures showed a depressed replication of both types of HSV. There was a borderline (P = 0.02) correlation between magnitude of HSV replication and presence of HL-A type B-w44, but this does not explain the HD control difference. Furthermore, the level of serum antibodies to HSV common antigen was not related to magnitude of in vitro replication. The results thus speak against generally enhanced cellular susceptibility to HSV as a reason for the high titres of serum antibodies to HSV in HD patients.


*Br. J. Cancer* (1981) **43**, 856

### *IN VITRO* SUSCEPTIBILITIES OF NORMAL HUMAN SKIN FIBROBLASTS TO ONCOVIRUSES, AND THE DECREASED SUSCEPTIBILITY TO HSV OF FIBROBLASTS FROM UNTREATED HODGKIN'S PATIENTS

P. EBBESEN†, B. F. VESTERGAARD‡, R. TING§, S. HAAHR||, J. GENNER¶ AND A. SVEJGAARD||

*From the †Institute of Cancer Research*, Radiumstationen, and the ||Institute of Medical Microbiology, DK-8000 Aarhus, the ‡Institute of Medical Microbiology, University of Copenhagen, and the ¶Rigshospitalet, DK-2100 Copenhagen, Denmark, and the §Biotech Research Laboratory, Rockville, Md, U.S.A.*

Received 7 July 1980 Accepted 9 March 1981

**Summary.—Fibroblast cultures established from the skin of 56 healthy controls and 15 untreated Stages I and II Hodgkin's patients (HD) were studied in their 3rd, 4th and 5th *in vitro* passage with respect to transformation with Simian sarcoma virus (SSV) and SV40 and with respect to replication of herpes simplex virus (HSV) Types 1 and 2, pox virus and interferon release. Susceptibility to the 5 viruses varied independently, except for an inverse correlation between susceptibility to SSV and HSV. HD cultures showed a depressed replication of both types of HSV. There was a borderline ($P=0{\cdot}02$) correlation between magnitude of HSV replication and presence of HL-A type B-w44, but this does not explain the HD control difference. Furthermore, the level of serum antibodies to HSV common antigen was not related to magnitude of *in vitro* replication. The results thus speak against generally enhanced cellular susceptibility to HSV as a reason for the high titres of serum antibodies to HSV in HD patients.**

HODGKIN'S DISEASE (HD) is associated with raised titres of serum antibodies to Epstein–Barr virus (EBV) (Levine *et al.*, 1971) to herpes simplex virus common antigen (Hesse *et al.*, 1977) and according to one publication also to Simian sarcoma virus gp70 (Ebbesen *et al.*, 1979). The relationship of these virological aberrations to aetiology and pathogenesis is unresolved; they might be primary or secondary to the changes in immune functions often detectable in HD, or HD might be characterized by an abnormal non-immunological relationship between virus and cells. The latter possibility was studied in the present work.

### MATERIALS AND METHODS

*Biopsies.*—A piece of abdominal skin was obtained from 56 controls, of which 33 were operated on for benign lesions such as large breast and 23 were completely healthy donors. The controls were 10–90 years of age (mean 31). Skin was also obtained from 16 Stages I and II HD patients during splenectomy for staging purposes. Their age varied from 8 to 70 years and 6 were classified as nodular sclerosis, 5 as lymphocyte predominance and 5 as mixed cellularity. The biopsies were immediately placed in cold Eagle's minimum essential medium with Hanks' balanced salt solution, pH 7·8 (MEM) with 10% foetal calf serum (FCS), which was also the medium for all fibroblast cultures. The biopsies had subcutaneous fat removed and were cut into pieces measuring $\sim 4\ mm^2$, which were placed in plastic bottles, barely covered with medium and left undisturbed for 3 days at 37°C in 5% $CO_2$. Thereafter change of medium was done thrice weekly for 3 weeks, before reseeding. All cultures were tested initially in the 3rd passage, in

* Sponsored by the Danish Cancer Society.

which the population doubling time in exponential phase was 72 h.

*Simian sarcoma virus.*—Simian sarcoma virus (SSV) (Theilen *et al.*, 1971) kindly supplied by Dr Fritz Dienhardt (Rush-Presbyterian-St Luke's Medical Centre, Department of Microbiology, Chicago, Illinois, U.S.A.) was cloned and harvested once from infected normal human skin fibroblasts, filtered (450 nm) and frozen. The titre in cells from person No. 53 was $5 \times 10^4$ FFU/ml. A fresh ampoule was used for each test. Similarly, a fresh vial from a frozen batch of normal cells from person No. 53 was thawed, cultured and included in each test. The day after seeding in small ($75cm^2$) Falcon plastic flasks, the subconfluent cultures with $\sim 10^5$ viable cells were infected. Half the cultures were pretreated with diethylaminoethyl-dextran (DEAE, 10 $\mu$g/ml) for 30 min and infected with one-tenth of the amount of virus used without DEAE. The virus doses used were 10 $\mu$l diluted 1:10 and 1:40. One-hit titration curves (transformed foci) were obtained with the most susceptible as well as the most resistant cells. All tests were done in triplicate at least twice.

*Herpes simplex virus (HSV) Type 1 and Type 2.*—Strain Macintyre and strain MS (American Type Culture Collection lot No. 1-D) were propagated in a rabbit cornea cell line. This cell line also served as cell control each time human fibroblasts were infected (Vestergaard *et al.*, 1972). The virus titres here were $2 \times 10^6 \pm 105$ PFU/ml for Type 1 and $3{\cdot}6 \times 10^6 \pm 105$ PFU/ml for Type 2. Cells harvested from the second *in vitro* passage of human fibroblasts were seeded in 60mm Petri dishes. At confluence ($5 \times 10^5$ cells) the cultures were infected with 10-fold-dilution rows ($10^{-1}$–$10^6$) of HSV and overlaid with maintenance medium containing 2% human serum with high-titre HSV antibodies for plaque development. As the same number of cells was present in each dish, the results obtained are plaques per $5 \times 10^5$ cells. For each test with 6 dilutions the titre was calculated by the Reed–Muench method. Rabbit cornea cells were included in each day's testing.

*Serum antibodies to HSV Type 1.*—Serum antibodies to HSV Type 1 were assessed in an ELISA assay using ion-exchange chromatographically purified antigen from Triton X-100 solubilized HSV Type 1 infected rabbit cornea cells (Vestergaard *et al.*, 1977).

*SV40 T-antigen assay.*—Two different preparations of small-plaque SV40 virus were used in the present study. They were grown in CV-1 cells and the titres were $1{\cdot}5 \times 10^8$ and $2 \times 10^8$ PFU/ml on CV-1 cells. Fibroblasts ($3$–$5 \times 10^5$) grown overnight in Falcon flasks were infected for 3 h at 37°C with small-plaque SV40 virus, at a multiplicity of infection of about 200. Infected cells were maintained in medium (RPMI 1640 with 10% foetal bovine serum) containing 1% rabbit anti-SV40 serum. Next day, cells were transferred to a square Petri dish containing 38-well printed slides in medium containing anti-SV40 serum. Forty-eight hours later, slides were washed with phosphate-buffered saline, fixed in cold acetone and stained for T-antigen by the indirect fluorescent-antibody method, using hamster T-antibody (courtesy of Dr K. Takemoto) and fluorescein-conjugated goat anti-hamster globulin. The number of T-antigen-positive cells in 2 different wells was counted. Cells were then stained with Giemsa and the total number of cells in each well was counted. The percentage of T-antigen-positive cells was then calculated (Takemoto *et al.*, 1968) and considered a marker for transformation. CV-1 cells were included in each day's testing.

*Pox virus.*—The vaccinia strain used for public vaccination in Denmark was used. Confluent cultures were washed $\times 3$ in PBS and inoculated with 50 haemagglutinating units of virus. After 30min incubation at 37°C, the cultures were again washed $\times 3$ and medium was added. The presence or absence of cytopathic effect was scored after 24, 48 and 95 h. Cells from person 53 were included in each day's testing.

*Statistics.*—The titres obtained with the various viruses (focus- or plaque-forming units/ml) were used as (inverse) measures of the susceptibility of the cultures. Correlation between values obtained with 2 different viruses was calculated by Spearman's rank correlation coefficient. Results obtained with cells from healthy donors were compared with those from HD cells by the non-parametric Mann–Whitney and $\chi^2$ tests.

*Induction of interferon.*—All cells used for induction of interferon were grown in 30ml culture flasks (Falcon®) in MEM supplemented with 15% FCS. After confluence, the cultures were washed and some cultures were given 5 ml MEM supplemented with 2% FCS containing Poly I:C (Miles Lab. code

11-231, lot 8) in different concentrations (*i.e.* 4·5, 0·5 and 0·05 u/ml). Other cultures were infected with 4 haemagglutinating units of Newcastle disease virus (NDV), drained after 2 h, washed with MEM and then given 5 ml MEM with 2% FCS. All cultures were incubated for 24 h, the medium was then harvested, dialysed against Sørensen buffer (pH 2) for 48 h at 4°C and then dialysed back to pH 7·4.

*Interferon assay.*—Determination of the antiviral activity was performed in cultures of human fibroblasts (Haahr, 1968). After growth to confluence in 60mm Petri dishes, the medium was replaced by 1 ml of 2-fold serial dilutions of specimens to be tested. Each dilution was tested in two cultures. After incubation for 6 h, the cultures were challenged with 50 PFU of vesicular stomatitis virus (VSV) strain Indiana. The cultures were drained after a 1h absorption and overlaid with 0·6% agar (DIFCO) in MEM. Neutral red was added 24 h later and the plaques were counted. Interferon titres were recorded as the reciprocal of the highest dilution which reduced the number of plaques counted in the controls by 50%. All assays were done ×3. Testing a stock preparation of interferon was included in each assay stock. Stock preparation of human fibroblast interferon was made by inoculating human skin fibroblast monolayers in 250ml Falcon bottles with 4 haemagglutinating units of NDV per cell suspended in 15 ml of MEM with 2% FCS. The supernatants were harvested after 24h incubation and dialysed against pH 2 for 48 h at +4°C and then dialysed back to pH 7·4. The titre was equivalent to 2000 iu of interferon per ml.

*HL-A typing.*—Simultaneously with the taking of skin biopsy, blood was drawn and part of it used for determining HL-A type; the remaining erythrocytes and granulocytes were removed on a Lymphoprep gradient (Bøyum, 1968) and were then frozen with 15% dimethylsulphoxide in a programmed freezer (1°C/min). HL-A typing was performed by the method of Kissmeyer-Nielsen and Kjerbye (1967). Patients and controls were typed for the following HL-A antigens: 1, 2, 3, 8, 10, 11, 25, 26, 28, 29, w19, w23, w24, w30+, w31, and w32 of the A series; 5, 7, 8, 12, 13, 14, 15, 16, 17, 18, 27, 37, 40, w16, w21, w22, w35, w38, w39, w41, w44, w45, and w47 of the B series; and w1, w3, and 24 of the C series.

### RESULTS

(1) Testing with our standard virus batches on our *standard cells* (person 53) included in each test gave these mean titres ± s.d. (focus-forming units (FFU), plaque-forming units (PFU) per ml): SSV $53 \pm 24 \times 10^3$ FFU, SSV+DEAE $112 \pm 29 \times 10^3$ FFU, HSV-1 $1{\cdot}98 \pm 0{\cdot}83 \times 10^6$ PFU, HSV-2 $3{\cdot}57 \pm 1{\cdot}3 \times 10^5$ PFU, SV40 $2{\cdot}6 \pm 1{\cdot}2\%$ stained cells. The interferon release from the standard cell was 8000 u/ml fluid medium after infection with NDV.

(2) Most of the results from testing cell cultures established from *healthy persons* are given in Fig. 1. In addition, Poly I:C gave detectable interferon release from only 5/40 tested, pox virus caused cytopathic effect within 72 h in 32/37 tested. Resistant cultures or permissive cultures remained so on testing 20 cultures during the 3 subsequent *in vitro* passages, and also after storage in liquid $N_2$ ($P < 0{\cdot}01$, $\chi^2$). From Day 1 to Day 5 after seeding, the cell population-doubling time was ~72h.

To study the kinetics of the SSV infection, response curves for 2 persons with high-permissive and 2 with low-permissive cultures were made (Fig. 2). There is some evidence of deviation from one-hit kinetics at low virus dose with the less susceptible cells.

SSV titre obtained with and without pretreatment with DEAE, correlated positively at the 0·001 level of probability, and SSV showed negative correlation with HSV-1 ($P < 0{\cdot}02$) (Fig. 3). Apart from this, no correlation between values for *in vitro* test with any 2 viruses, or between *in vitro* virus titre and level of antibodies to HSV in serum, was found, and no correlation with those values and the NDV-provoked interferon activity (Table). The titres obtained were also uncorrelated to HL-A type ($P > 0{\cdot}05$), except for an apparent correlation ($P = 0{\cdot}02$) between high susceptibility to HSV-1 and HSV-2 and the presence of the antigen B-w44. The HD fibroblasts most resistant to HSV-1 and/or HSV-2 were of the histo-

FIG. 1.—Foci or plaques obtained by infecting human skin fibroblasts from healthy donors. Units of abscissae are respectively: SSV, 10 foci. SV40, 0·1% on left; 1% on right. Interferon, $2 \times 10^3$ units. HSV 1 and 2, one tenth the plaques obtained with standard cells. Anti-HSV, one tenth of standard optical density.

logical subtype lymphocyte predominance and mixed cellularity (not statistically significant at the 1% level).

(3) Comparison of virus susceptibility between fibroblasts from *HD patients and healthy persons* was made by calculating the mean FFU or PFU values obtained with the standard cells as 100. The mean values found in fibroblasts from healthy persons and HD patients were thereafter

FIG. 2.—Dose-response curves for infection of human skin fibroblasts from 4 normal donors with SV40. 6 cultures were made from each donor for each virus dilution. 10 μl of virus dilution/75 $cm^2$ flask with $10^5$ fibroblasts. Vertical bars indicate standard deviation. ----- susceptible cultures from donor 1. —— susceptible cultures from donor 2. -- -- non-permissive cultures from donor 3. ····· non-permissive cultures from donor 4.

expressed in relation to the standard values obtained on standard cells, as shown in the Table. It is seen that the mean value

TABLE.—*Mean* in vitro *FFU or PFU obtained with 5 viruses, and interferon releases after ND virus infection of skin fibroblast cultures from normal and HD patients*

| Virus | Cells | Mean ± s.d. |
|---|---|---|
| SSV with DEAE | Normal | 64 ± 35 |
| | HD | 84 ± 43 |
| SSV without DEAE | Normal | 89 ± 49 |
| | HD | 119 ± 47 |
| HSV-I | Normal | 24 ± 6 |
| | HD | 13 ± 7 |
| HSV-II | Normal | 107 ± 32 |
| | HD | 69 ± 20 |
| SV40 | Normal | 99 ± 57 |
| | HD | 87 ± 55 |
| Interferon release | Normal | 101 ± 63 |
| | HD | 71 ± 44 |

100 = value obtained with standard cells in each test.

with and without DEAE-dextran pretreatment of the cells (100 = value with a standard normal cell whether DEAE-dextran pretreated or not) was 84 and 100 in HD and 64 and 89 in control cultures. Also, for SV40 and Vac virus no difference was discernible (Vaccinia virus caused cytopathic effect within 72 h in 18/20 HD cultures), but both HSV-1 and HSV-2 gave lower values in HD than in controls ($P < 0{\cdot}05$, Table I). *This titre difference persisted in the 3 subsequent cell passages.* Matching patients and controls according to age and sex did not change the outcome of the comparison.

The HL-A antigen frequencies in the controls correspond to those previously

FIG. 3.—Ordinate; HSV type 1-induced plaques. Abscissa; SSV-induced foci on the same persons' fibroblast culture. 100 units on each axis is the response with standard cells.

established in healthy Danes. No correlation was discernible between HL-A type and *in vitro* susceptibility to infection with any of the viruses tested, or to titre of serum antibodies to HSV with cells from HD patients.

DISCUSSION

Resistance to viral infection can operate by 2 basic mechanisms. First, the resistance may reflect an accelerated immune response. The second type of resistance resides in the target cells. It usually persists in tissue culture, though cultured cells can have a spectrum of sensitivity to viral infection broader than that of the organ from which they came (Evans *et al.*, 1954). However, *in vitro* studies are the only possibility for elucidating this aspect of human resistance.

As we found that cultures from one healthy individual could be relatively resistant to one virus and at the same time fully susceptible to another, no common non-specific mechanism can be held responsible for the differences in susceptibility among individuals. It is in line with this fact that growth rate *in vitro* varied too little to account for differences in *in vitro* susceptibility to virus, and that no correlation to interferon production after stimulation with NDV or POLY I:C was apparent; again, the interferon production may have a different pattern with each virus (de Mayer *et al.*, 1974).

Susceptibility to the 5 viruses tested varied independently, the exceptions being the inverse relationship between susceptibility to SSV and susceptibility to HSV-1 and HSV-2, which suggests the possibility that infection with SSV and HSV partly depends on mutually exclusive cell characteristics.

The genetic factors that can influence host-virus interactions are numerous. One such of proven relevance in the intact individual is the Ir genes controlling specific immune response (Spencer *et al.*, 1976). Some Ir genes are linked to the major histocompatibility system; others are not (Old & Boyse, 1972). But since neither the blood types nor HL-A types tested correlated with *in vitro* susceptibility, the evidence is that expression of these antigens is not linked to expression of factors important for *in vitro* susceptibility. *In vitro*-grown chick fibroblasts vary in adsorption of C-type virus (Vogt & Ishizaki, 1965) whereas the resistance of mouse fibroblasts to certain C-type viruses resides in intracellular events (Bassin *et al.*, 1975) as does cellular resistance to vesicular stomatitis (Simpson & Obijeski, 1974). Non-permissiveness, of course, could also reflect true interference from pre-existing infection of the cells.

HL-A type A1 shows increased incidence among healthy persons with recurrent herpes labialis (Russell & Schlaut, 1975). Among the 60 persons here tested, no correlation exists between HL-A type and titre of antibody to HSV. The link between HL-A type A and *Herpes labialis* therefore seems to depend upon other genes than those controlling the humora immune response to HSV. Cellular immunity is believed to be most important for immune resistance to HSV (Rager-Zisman & Allison, 1976; Shore *et al.*, 1974). The lifetime persistence in most adult humans of high titres of antibodies to HSV probably results from nearly all adults being chronic virus carriers. The adults with persistently low antibody titres to HSV could therefore be persons effectively eliminating emerging virus by their cellular immune response, or they might have non-permissive cells. Our results do not support the latter possibility, as no significant correlation between individual *in vitro* susceptibility and level of antibody to HSV was discernible.

Our finding that cultures from untreated *Hodgkin's patients* are less susceptible to *in vitro* infection with HSV (both Types 1 and 2) than normal cultures in spite of no significant differences in susceptibility to SSV, SV40 and Vac, suggests that Hodgkin's cultures are indeed characterized by an abnormal relationship to herpes-type virus, and that the result does not represent a fortuity in our multiparameter study. As early-passage cultures, not cloned cell lines, were studied here, we do not know whether the difference found between HD and controls reflects characteristics common to most of an individual's fibroblasts or differences in proportion of susceptible and non-susceptible cells. A few cloning experiments were inconclusive on this point. It is noticeable that most (Levine *et al.*, 1971; Henderson *et al.*, 1973; Hesse *et al.*, 1977) but not all (Evans *et al.*, 1978) studies show elevated titres of serum antibody to members of the herpes virus group in HD patients, whereas these patients have normal titres of antibodies to influenza (Ebbesen *et al.*, 1979) and adenovirus (Hesse *et al.*, 1977).

Both the aetiology and pathogenesis of Hodgkin's disease are unresolved, but it is usually considered primarily a disease of the lympho-reticuloendothelial cells, sometimes with secondary stimulation of growth of other cell types such as fibroblasts (Wahl *et al.*, 1978; Kaplan *et al.* 1977). If, indeed, the isolated HD fibroblasts differ from normal fibroblasts as indicated by our study, 2 more possibilities arise. Either the changes in the reticuloendothelial system are secondary to some (virus induced?) alteration in fibroblasts, or HD represents the expression of pleiotropic regulatory gene changes which also give the decreased *in vitro* susceptibility to herpes virus.

The association of HL-A type with disease is particularly pronounced in certain states with altered immune reactivity (Svejgaard *et al.*, 1976). Immune regulatory Ir genes are in certain cases associated more firmly with diseases than is the HL-A system (Wernet, 1976), but the Ir genes are themselves closely linked to the HL-A system. HL-A antigens are preserved during *in vitro* growth of human fibroblasts (Brautbar *et al.*, 1973). The number of cases we have studied so far, however, do not allow conclusion as to whether *in vitro* susceptibility of HD fibroblasts to the virus tested is HL-A related.

This investigation was supported by the Danish Cancer Society, the Danish Medical Research Council, Daell Fonden, P. Carl Pedersend Fond, R. Rask-Nielsens Fond and Fonden til Lægevidenskabens Fremme.

### REFERENCES

Bassin, R., Gerwin, B. E., Duran-Troise, G. & Gissel-Brecht, S. (1975) Murine sarcoma virus pseudotypes acquire a determinant specifying N or B tropism from leukemia virus during rescue. *Nature*, **256**, 223.

Brautbar, C., Pellegrino, M. A., Ferrone, S., Reisfeld, R. A., Payne, R. & Hayflick, L. (1973) Fate of HL-A antigens in aging cultured human diploid cell strains. *Exp. Cell Res.*, **78**, 367.

Böyum, A. (1968) Separation of leukocytes from blood and bone marrow. *Scand. J. Clin. Lab. Invest.*, **21** (Suppl. 97), 91.

Ebbesen, P., Due, C., Hesse, J. & 4 others (1979) Elevated titre of antibodies to Simian sarcoma virus envelope antigen (gp70) and normal response to influenza virusi n untreated Danish Hodgkin's patients. *Int. J. Cancer*, **24**, 1.

Evans, A. S., Carvalho, R., Frost, P., Famra, M. & Possi, D. (1978) Epstein–Barr virus infection in Brazil. II. Hodgkin's disease. *J. Natl Cancer Inst.*, **61**, 19.

Evans, C. A., Chambers, V. C., Smith, W. H. & Byatt, P. H. (1954) Growth of neurotropic viruses in extraneous tissues: Survey of tissues of various animal species for capacity to support multiplication of poliomyelitis virus *in vitro*. *J. Infect. Dis.*, **94**, 273.

Haahr, S. (1968) The occurrence of interferon in the cerebrospinal fluid in patients with bacterial meningitis. *Acta Path. Microbiol. Scand.*, **74**, 445.

Henderson, B. E., Dworsky, R., Mench, H. & 4 others (1973) Case-control study of Hodgkin's disease. II. Herpesvirus group antibody titers and HL-A type. *J. Natl Cancer Inst.*, **51**, 1443.

Hesse, J., Levine, P., Ebbesen, P., Conelly, R. R. & Mordhorst, C. H. (1977) A case control study on immunity to two Epstein–Barr virus-associated antigens, and to herpes simplex virus and adenovirus in a population-based group of patients with Hodgkin's disease in Denmark, 1971–73. *Int. J. Cancer*, **19**, 49.

Kaplan, H. S., Goodenow, R. S., Epstein, A. L., Gartner, S., Decleve, A. & Rosenthal, P. H. (1977) Isolation of a type C RNA virus from an established human histiocytic lymphoma cell line. *Proc. Natl Acad. Sci. U.S.A.*, **74**, 2564.

Kissmeyer-Nielsen, F. & Kjerbye, K. E. (1967) Lymphocytotoxic micro-technique: Purification of lymphocytes by flotation. In *Histocompatibility Testing 1967*. Ed. Curtoni *et al.* Copenhagen: Munksgaard. p. 381.

Levine, P. H., Ablashi, D. V., Berard, C. W., Carbone, P. P., Waggoner, D. E. & Malan, L. (1971) Elevated antibody titers to Epstein–Barr virus in Hodgkin's disease. *Cancer*, **27**, 416.

de Mayer, E., de Mayer-Guignard, W., Hall, T. & Bailey, D. W. (1974) A locus affecting circulating interferon levels induced by mouse mammary tumor. *J. Gen. Virol.*, **23**, 209.

Old, L. & J. Boyse, E. A. (1972) Current enigmas in cancer research. *Harvey Lect.*, **67**, 273.
Rager-Zisman, B. & Allison. A. C. (1976) Mechanisms of immunologic resistance to herpes simplex virus 1 (HSV-1) infection. *J. Immunol.*, **116**, 35.
Russell, A. S. & Schlaut, J. (1975) HL-A transplantation antigens in subjects susceptible to recrudescent herpes labialis. *Tissue Antigens*, **6**, 257.
Shore, S. L., Nahmias, A. J., Starr, S. E., Wood, P. A. & McFarlin, D. E. (1974) Detection of cell-dependent cytotoxic antibody to cells infected with herpes simplex virus. *Nature*, **251**, 351.
Simpson, R. W. & Obijeski, J. F. (1974) Conditional lethal mutants of vesicular stomatitis virus. I. Phenotypic characterization of single and double mutants exhibiting host restriction and temperature sensitivity. *Virology*, **57**, 357.
Spencer, M. J., Cherry, J. D. & Terasaki, P. F. (1976) HL-A antigens and antibody response after influenza A vaccination. Decreased response associated with HL-A type w16. *N. Engl. J. Med.*, **294**, 13.
Svejgaard, A., Hauge, M., Jersild, C. & 4 others (1976) The HLA system. An introductory survey. In *Monographs in Human Genetics*, Vol. 7. Basel: Karger. p. 1.
Takemoto, K. K., Ting, R., Ozer, H. L. & Fabisch, P. (1968) Establishment of a cell line from an inbred mouse strain for viral transformation studies: Simian virus 40 transformation and tumor production. *J. Natl Cancer Inst.*, **41**, 1401.
Theilen, G. H., Gould, D., Fowler, M. & Dungworth, D. L. (1971) C-type virus in tumor tissue of a Woolly monkey (*Lagothrix* sp.) with fibrosarcoma. *J. Natl Cancer Inst.*, **47**, 881.
Vestergaard, B. F., Grauballe, P. C. & Spanggaard, H. (1977) Titration of herpes simplex virus antibodies in human sera by the enzyme-linked immunosorbent assay (ELISA). *Acta Pathol. Microbiol. Scand.* (Sect. B), **85**, 446.
Vestergaard, B. F., Hornsleth, A. & Pedersen, S. N. (1972) Occurrence of herpes and adenovirus antibodies in patients with carcinoma of the cervix uteri. *Cancer*, **40**, 68.
Vogt, P. K. & Ishizaki, R. (1965) Reciprocal patterns of genetic resistances to avian tumor viruses in two lines of chickens. *Virology*, **26**, 664.
Wahl, S. M., Wahl, L. M. & McCarthy, J. B. (1978) Lymphocyte-mediated activation of fibroblast proliferation and collagen production. *J. Immunol.*, **121**, 942.
Wernet, P. (1976) Human Id-type alloantigens: Methods of detection, aspects of chemistry and biology, markers for disease states. *Transplant. Rev.*, **30**, 271.